# Extraction of His Bundle Pacing Lead: More Difficult than Coronary Sinus Lead Extraction: An Analysis of 3897 Lead Extraction Procedures Including 27 His and 253 Coronary Sinus Lead Removals

**DOI:** 10.3390/biomedicines12061154

**Published:** 2024-05-23

**Authors:** Paweł Stefańczyk, Wojciech Jacheć, Andrzej Kutarski, Paweł Dąbrowski, Andrzej Głowniak, Dorota Nowosielecka

**Affiliations:** 1 Department of Cardiology, The Pope John Paul II Province Hospital of Zamość, 22-400 Zamość, Poland; paolost@interia.pl (P.S.); paveldabrowski@interia.pl (P.D.); 2 2nd Department of Cardiology, Faculty of Medical Sciences in Zabrze, Medical University of Silesia, 40-055 Katowice, Poland; wjachec@interia.pl; 3 Department of Cardiology, Medical University of Lublin, 20-093 Lublin, Poland; a_kutarski@yahoo.com (A.K.); andrzej.glowniak@gmail.com (A.G.); 4 Department of Cardiac Surgery, The Pope John Paul II Province Hospital of Zamość, 22-400 Zamość, Poland

**Keywords:** His bundle pacing lead extraction, lead extraction in conduction system pacing, coronary sinus lead extraction, lead extraction complexity

## Abstract

Background: Experience with the transvenous extraction of leads used for His bundle pacing (HBP) is limited. Methods: Analysis of 3897 extractions including 27 HBP and 253 LVP (left ventricular pacing) leads. Results: The main reason for HBP lead extraction was lead failure (59.26%). The age of HBP and LVP leads (54.52 vs. 50.20 months) was comparable, whereas procedure difficulties were related to the LVP lead dwell time. The extraction of HBP leads > 40 months old was longer than the removal of younger leads (8.57 vs. 3.87 min), procedure difficulties occurred in 14.29%, and advanced tools were required in 28.57%. There were no major complications. The extraction time of dysfunctional or infected leads was similar in the HBP and LVP groups (log-rank *p* = 0.868) but shorter when compared to groups with other leads. Survival after the procedure did not differ between HBP and LVP groups but was shorter than in the remaining patients. Conclusions: 1. HBP is used in CRT-D systems for resynchronisation of the failing heart in 33.33%. 2. Extraction of HBP leads is most frequently performed for non-infectious indications (59.26%) and most often because of lead dysfunction (33.33%). 3. The extraction of “old” (>40 months) HBP leads is longer (8.57 vs. 3.87 min) and more difficult than the removal of “young” leads due to unexpected procedure difficulties (14.29%) and the use of second line/advanced tools (28.57%), but it does not entail the risk of major complications and procedure-related death and is comparable to those encountered in the extraction of LVP leads of a similar age. 4. Survival after lead extraction was comparable between HBP and LVP groups but shorter compared to patients who underwent the removal of other leads.

## 1. What’s New?

Conduction System Pacing (CSP) delivered by His Bundle Pacing (HBP) or Left Bundle Pacing (LBP) appears to compete with biventricular pacing but may be of limited durability due to more frequent pacing/sensing failures than in other leads. Transvenous lead extraction is of key importance in the proper management of dysfunctional or infected leads. The lumenless Medtronic Select Secure 3830 lead is most popular for CSP, but experience with such lead extraction is very limited, especially if the leads are older than 4 years. This study presents a broad analysis of data to compare the difficulty, complexity, and complications of CSP and LVP lead extraction.

## 2. Introduction

Conduction system pacing (CSP) delivered by His Bundle Pacing (HBP) or Left Bundle Pacing (LBP) is a relatively new development in the field of more physiological pacing and cardiac resynchronisation therapy (CRT). Although the concept of HBP was created 23 years ago [1], it was widely introduced only a few years ago [2,3,4,5,6,7,8] both as an option for the resynchronisation of a failing heart [2,9] and as a non-desynchronising option for ventricular pacing in patients without indications for CRT [3,4,5,6,7,8]. Between 2018 and 2021, HBP was the most prevalent choice [2,3,4,5,6,7,8,9], and since 2019, it has been gradually replaced by LBP [10,11,12,13,14]. The effectiveness of CPS device implantation is 67–92% [1,3,7], whereas the durability of HBP is shorter than that of other types of cardiac pacing. The rate of dysfunctional HBP leads was 14.3% in the initial period [1], and now, it has ranged from 4.3 to 12.1% over a period of several years [2,3,4,5,6,7], but in the longer term, it increases to 53% [8]. It is expected that with the increasing popularity of this type of system, there will be more problems in the long-term follow-up of patients with CSP [15]. Transvenous lead extraction (TLE) is the first-line option for the management of dysfunctional, infected, and abandoned leads [16]. In most patients referred for CSP, the thin lumenless Medtronic Select Secure 3830 lead has been used, originally intended for children for whom its small diameter had a clear advantage over standard leads [15]. Experience with the extraction of these leads in 33 children after several (2–5) years of operation has been described [17,18,19]. However, our understanding of CSP lead extraction is very limited, as there is only one report presenting 30 extractions of HBP leads [20], one case series (nine cases) [21], and two case reports [22,23]. The lead implant duration in the cited studies was 25 months or significantly less, except one HBP lead with a dwell time of 14.3 years [22]. The knowledge of LBP lead extraction is even more modest, as it is based on six case reports [24,25,26,27,28,29].

## 3. Goal of the Study

The goals of this study were as follows: 1. to analyse the clinical and technical aspects of HBP lead extraction, including the influence of the lead dwell time on the effectiveness and complications of TLE; 2. to compare the difficulty and complexity of HBP and conventional LVP lead extraction, as well as survival after TLE in these groups.

## 4. Methods

### 4.1. Study Population

Data from 3897 transvenous lead extraction (TLE) procedures (27, HBP group; 253, LVP group; 3617, control group; TLE of other than HBP or LVP leads) performed between March 2006 and May 2023 at a single high-volume centre were reviewed.

### 4.2. Lead Extraction Procedure

#### 4.2.1. Definitions

Indications for lead removal, procedure effectiveness, and complications were defined according to the lead management recommendations (2017 HRS consensus and 2018 EHRA guidelines) [16,30]. The risk of major complications (MCs) related to TLE was assessed using the SAFeTY TLE score, an online tool available at http://alamay2.linuxpl.info/kalkulator/ and http://usuwanieelektrod.pl/kalkulatory (accessed on 28 January 2020) [31].

The EROS score was used for the prediction of significant procedural complications that required emergent surgical intervention [32]. The assessment of procedure complexity was based on the MB—Mazzone-Bontempi (score), showing the need for the use of advanced tools to achieve TLE success [33], LED—Lead Extraction Difficulty index referring to lead extraction difficulty based on fluoroscopy times [34], Advanced LE Techniques (ALET) score to predict the necessity of using advanced extraction techniques [35], and the Complex Indicator of the Difficulty of the TLE (CID-TLE) and LECOM score based on time of lead extraction and use of metal sheath or Evolution/TightRail, lasso-catheters, or basket catheters or the use of an alternative approach [36].

Unexpected difficulties during the extraction procedure, so-called “technical problems”, were defined as the situations that increased the complexity of the procedure but were not complications [37].

#### 4.2.2. Techniques of HBP Lead Extraction

**Figure 1:**
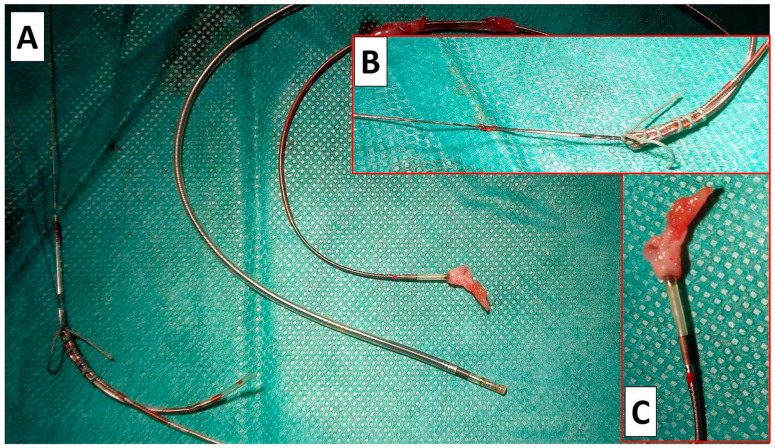
Our “home-made” semi-rigid lead extender using a standard stylet and ligature. Example of the removed HBP leads. General view (**A**). Extension of lumenless HBP lead with a sharp-angled standard stylet and garter. The system enables easy slipping of a polypropylene sheath onto the lead (**B**). Scar tissue on ended removed HBP lead (**C**).

**Figure 2:**
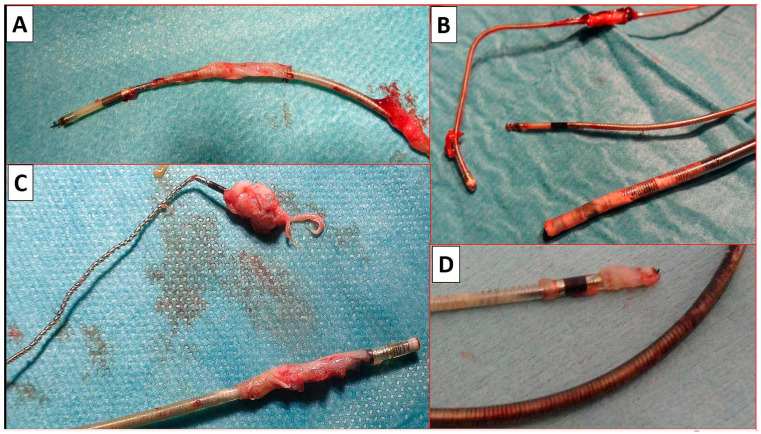
Examples of removed HBP leads with implant durations > 4 years. Scar tissue (**A**–**D**) is seen on all removed HBP leads. The scarring sometimes stays in place (**A**,**B**,**D**) and sometimes slides off towards the tip of the lead (**C**).

The Medtronic Select Secure 3830 lumenless (Medtronic, MN, USA) lead was developed for use in the paediatric population due to its small diameter. Later, it proved to be excellent also for CSP. The lead is characterised by an exposed nonretractable helix [15]. These features affect the technical aspects of the TLE procedure. The impossibility of introducing a locking stylet requires the use a semi-rigid lead extender, e.g., Bulldog System (Cook Medical, Bloomington, IN, USA) [15,19,22]. Although paediatric experience has shown that a significant percentage of leads can be removed through manual traction alone [17,18,19], this technique was used only in patients with device infection. In the present study, most of the extraction procedures were performed to replace a dysfunctional lead; therefore, we tried to maintain the original venous access. As a rule, we used polypropylene sheaths of the smallest possible diameter to reduce the risk of dislodgement of the remaining functional leads. For practical reasons, we used a sharply bent standard stylet and a fixation ligature to extend the lead (Figure 1 and Figure 2).

It should be noted that in the case of the short age of the leads (<40 months), the problem was rather too early disruption of the lead from scar tissue, which could result in the accidental removal of the lead and loss of venous access. Therefore, when the tip of the lead was freed after gentle traction, we usually held it with a lasso inserted from the femoral access. This made it possible to obtain a safe “rail” effect for the polypropylene sheath [15,21]. In only a few cases, it was necessary to replace the sheath with a new one with a larger diameter, and Evolution had to be used only when we preferred to extract leads using non-powered mechanical polypropylene sheaths, bearing in mind that the remaining functional leads should be kept untouched if not planned to be removed (Figure 2).

### 4.3. Dataset and Statistical Methods

#### 4.3.1. Creation of Subgroups for Analysis

**Figure 3:**
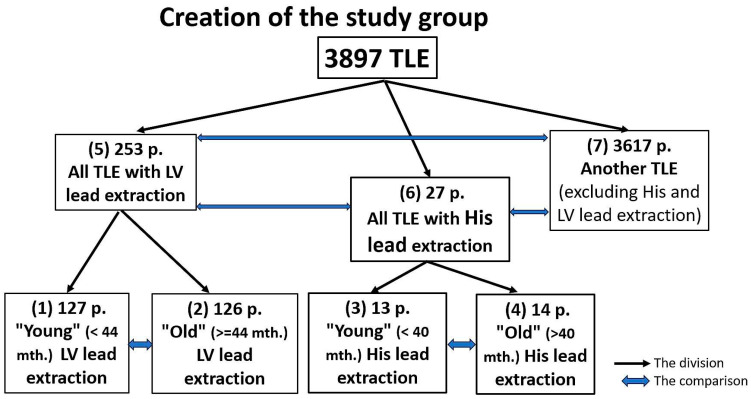
Creation of the study groups. TLE—transvenous lead extraction, LV (pacing) lead—a lead designed for pacing of the left ventricle.

Figure 3 illustrates how the study participants were divided into groups. The division of the study cohort into groups is presented in Figure 3. Groups 5, 6, and 7 were identified to assess the difficulty and efficiency of HBP and LVP lead extraction.

Groups 1, 2, 3, and 4 were identified to investigate the effect of the HBP and LVP lead implant duration on the difficulty and efficiency of the extraction procedure. HBP and LVP subgroups were selected based on the median age of the electrodes (HBP, 40 months; LVP, 44 months) (Figure 3).

#### 4.3.2. Statistical Analysis

Continuous variables are presented as the mean ± standard deviation. The categorical variables are presented as counts and percentages. The significance of differences between the groups was determined using the non-parametric Chi2 test with Yates correction or the unpaired Mann–Whitney U test, as appropriate. The Bonferroni correction was applied for a comparison among groups 6, 5, and 7 (considering a *p*-value < 0.0166 as statistically significant). To determine the effect of HBP and LVP leads on the survival of the CIED system and survival of patients after TLE, Kaplan–Meier curves were plotted, and differences in their course were assessed using the log rank test. A *p*-value less than 0.05 was considered statistically significant. Statistical analysis was performed with Statistica 13.3 (TIBCO Software Inc., Tulsa, OK, USA).

#### 4.3.3. Approval of the Bioethics Committee

All patients gave their informed written consent to undergo TLE and use anonymous data from their medical records, approved by the Bioethics Committee at the Regional Chamber of Physicians in Lublin no. 288/2018/KB/VII (approval date: 27 November 2018). The study was carried out in accordance with the ethical standards of the 1964 Declaration of Helsinki.

## 5. Results

In the HBP lead group consisting of 27 patients with a median of age of 70.52 years, there were 29.63% women, and 40.74% of the procedures were performed for infectious reasons. In the LVP lead group consisting of 253 patients with a median of age of 68.87 years, there were 20.95% women, and 62.05% of the procedures were performed for infectious reasons. In the remaining 3.617 patients (extraction of other than HBP and LVP leads) with an average age of 65.84 years, there were 39.37% women, and 29.08% of the procedures were performed for infectious reasons (Table 1).

Generally, the HBP and LVP groups, as well as their subgroups with “old” and “young” leads, did not differ from each other, i.e., they were made up of very similar populations. The HBP patients had a slightly higher LVEF than the LVP patients because HBP was not used to resynchronise the failing heart in some patients. Obviously, the control group had a higher LVEF and a significantly lower rate of a worse NYHA class than the patients with HBP and LVP leads. The complexity of the systems and multiple previous CIED-related procedures increased the risk of systemic infection expressed as the PADIT score, more in patients with LVP than HBP leads compared to the control group. Patients with older LVP leads were more likely to have mechanical lead damage and dysfunction, and this phenomenon was not observed in those with HBP leads (Table 1).

HBP leads were used more often in standard pacing systems (66.67%) than in CRTD systems (33.33%) with the opposite order for left ventricular leads, but in that case, the LVP lead was a component of the CRT-P system. Although the mean age of the oldest lead removed in the “old” HBP and LVP subgroups was similar to the age of the oldest lead removed in the control group (105.9, 90.74 and 103.5 months, respectively), the age of the oldest lead removed per patient in the HBP and LBP groups was lower than that in the control group (74.81, 68.12, and 103.5 months, respectively). The lead (HBP and LVP) implant duration was similar (54.52 and 50.20 months). Patients with systems containing HBP and LVP leads had more leads in the heart (2.52, 3.04 vs. 1.87) and more procedures (1.92, 2.09 vs. 1.84) before TLE than patients in the control group (Table 2).

Scores of risk factors for major complications or procedure complexity indicated greater difficulty and increased risk during the extraction of older leads in the HBP and LVP subgroups (SAFeTY LE, MB, LED, ALET, LECOM), but only the LECOM score indicated an increase in procedure complexity in the case of LVP versus HBP leads (27.09 vs. 19.41%). The LVP group was characterised by a higher rate of CIED-related procedures and a higher rate of removed passive fixation leads (excluding LV leads). This was due to historical reasons (in earlier years, LVP was used significantly more often than HBP) (Table 2).

The extraction duration was longer in the “old lead” subgroups, while the lead extraction time did not differ significantly between the HBP and LVP lead groups and the control group. The extraction of a single HBP/LVP lead was twice as long in the subgroups with “old” leads, while it did not differ in relation to the entire HBP and LVP groups (3.19 vs. 3.05 min). In 85.19% and 70.75% of patients, HBP and LVP leads were removed using mechanical dilation and when interpreting these percentages, it should be borne in mind that, respectively, 59.26% and 37.94% of the procedures were performed for non-infectious indications with the aim to replace the lead for a new one while maintaining the original venous access. Gentle screw-out and gentle traction to remove the HBP and LVP lead were mainly used in patients with device infection (14.81 and 28.97% respectively) (Table 3).

The procedure difficulty was similar in all the study groups and subgroups. The values of the CID-TLE score were higher in the subgroups with old leads but did not differ significantly among the HBP, LVP, and control group. Similarly, there were no differences in the number of more complex procedures (2 and more CID score points) (18.52, 28.06, and 18.30%, respectively).

Rates of major complications (MCs), rescue cardiac surgery, and procedure-related deaths were very low and did not differ between the study groups. Due to a higher rate of partial radiographic success (retained tip or <4 cm lead fragment) in the “old” LVP lead subgroup (more passive fixation leads removed), the rate of procedural success was slightly lower.

The FU period in survivors was shorter for patients with removed HBP leads, as this technique was introduced significantly later than LVP (Table 3).

The presence of an ICD lead (CRTD) before TLE was the only risk factor for death in patients undergoing the removal of His pacing leads (Table 4).

**Figure 4:**
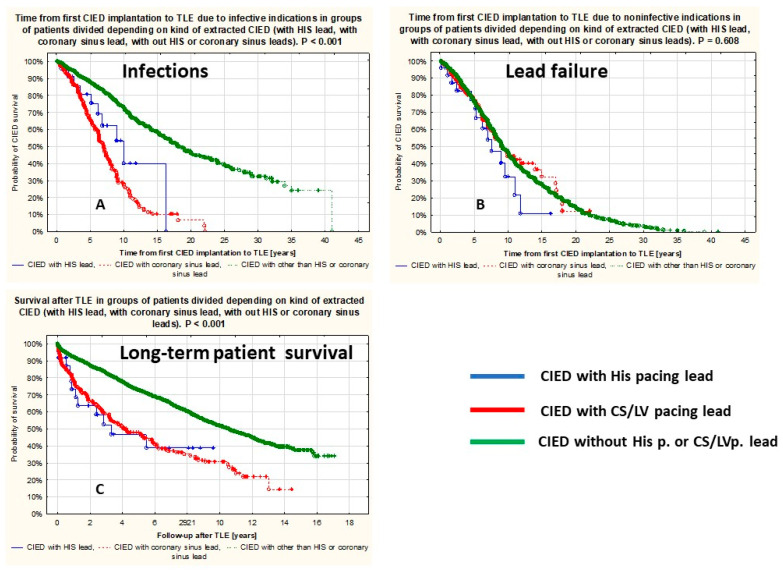
Lead survival and patient survival. Lead survival—time since lead implantation to extraction due to infection (**A**), lead failure (**B**), and patient survival after CIED extraction (**C**).

The time from implantation to TLE for non-infectious indications did not differ between the groups (log rank *p* = 0.868), and the time to TLE for infectious indications was comparable in the HBP and LVP groups and was shorter when compared to the group with other leads. Survival after TLE did not differ between HBP and LVP groups but was lower when compared to the group with other leads (Figure 4).

## 6. Discussion

The haemodynamic benefits of conduction system pacing (CSP) have long been known [1], but the intervention has become increasingly popular only in recent years [2,3,4,5,6,7,8,9]. The use of this type of physiological ventricular pacing has been extensively described in various situations: in patients with a normal heart to prevent post-pacing cardiomyopathy (bradycardia indications) [1,3,4,5,6,7,8,15], as a method of ventricular resynchronisation in patients with heart failure and wide QRS complexes [2,3,5,7,15], and as a component of resynchronisation systems (CRT-D), in which the electrode stimulating the conduction system (HBP, LBP) is used for resynchronisation and the ICD electrode is meant for high-voltage therapy only [5,9,15], a dysfunctional left ventricular transvenous pacing lead is replaced by a CSP lead [15] or implanted when LVP lead implantation fails [9,15].

In recent years, several limitations of HBP have been recognised, i.e., an unacceptable increase in the pacing threshold or a decrease in the sensed ventricular potential values, which makes a dozen or more percent of the leads cease to fulfil their role within a few years [1,2,3,4,5,6,7,8]. Today, the seemingly less sophisticated LBP lead guarantees better long-term performance, values of the sensed ventricular potential are much higher, the risk of early dislodgement is practically non-existent, and the pacing threshold values increase less frequently (in 8%) [15]. At present, we have an increasing population of patients with HBP leads implanted several years ago, and the demand for LBP lead replacement is likely to grow in the coming years [15]. The difficult technique of implanting leads into the CSP prolongs the implantation procedure, which increases the risk of infectious complications. The basic component of optimal lead management is transvenous lead extraction [16], and the odds are that the interest in CSP lead extraction techniques will increase dramatically.

Thin lumenless Select Secure 3885 leads are commonly used for CSP. Experience with their extraction comes from the paediatric population (34 children, lead extraction a few years after implantation), and three reports showed that 41–100% can be removed through manual traction [17,18,19]. The knowledge and experience of the extraction of CSP leads is very limited as it comes from one report of 30 HBP lead extractions [20], one case series (9 cases) [21], and 2 case reports [22,23] and one review paper [15]. Of the 41 HBP lead removals, mechanical dilatation was necessary in 10 patients (24.4%) [20,21,22,23]. There is no study on LBP lead extraction, apart from 6 case reports. There is no available literature on extraction failure or rupture of the Select Secure 3880 lead during extraction.

This study showed that the EF and NYHA class in the HBP group were better than in the LVP group. Patients with HBP and LVP had more leads in the heart and more CIED-related procedures before TLE than patients in the control group. The age of extracted HBP or LVP leads did not differ significantly (54.52 vs. 50.20 months). The extraction of “old” (>40 months) HBP leads was longer than the removal of “young” (<40 months) HBP leads (8.57 and 3.87 min). Mechanical dilatation was necessary in 69.23% and 100% of HBP leads, but it should be borne in mind that in 35.71% and 46.15%, respectively, the procedures had been performed for non-infectious indications with the intention to replace the lead for a new one while maintaining the original venous access. Unexpected procedure difficulties (“technical problems) appeared in 14.29%, and second line/advanced tools were used in 28.57% during the extraction of “old” HBP leads only. Difficulties in extracting HBP leads were comparable to those encountered in the extraction of LVP leads of similar age. Consistent with the cited reports [20,21,22,23] in this study, there were no major complications, rescue cardiac surgery, or procedure-related death during HBP and LVP lead extraction. The follow-up duration was shorter in patients with removed HBP leads, as this technique was introduced much later than LVP. Kaplan–Meier analysis showed comparable survival of patients with LVP and HIS lead extraction; however, it was shorter than in the control group.

## 7. Conclusions

1. HBP is used in CRT-D systems for resynchronisation of the failing heart in 33.33%.
2. The extraction of HBP leads is most frequently performed for non-infectious indications (59.26%) and most often because of lead dysfunction (33.33%).
3. The extraction of “old” (>40 months) HBP leads is longer (8.57 vs. 3.87 min) and more difficult than the removal of “young” leads due to unexpected procedure difficulties (14.29%) and the use of second line/advanced tools (28.57%), but it does not entail the risk of major complications and procedure-related death and is comparable to those encountered in the extraction of LVP leads of a similar age.
4. Survival after lead extraction was comparable between HBP and LVP groups but shorter compared to patients who underwent the removal of other leads.

### Study Limitations

All procedures were performed using all types of mechanical systems but not laser-powered sheaths. The study aimed to assess the effectiveness and outcomes of HBP and LVP lead extraction. But complexity and major complications apply to the entire procedure and not to the extraction of one type of the lead(s). As removal of the HBP and LVP leads was often accompanied by the extraction of other pacemaker/ICD leads, we could never be sure which of these leads caused the complication. Patients with HBP and LVP leads often had abandoned leads or newer leads implanted during upgrade procedures. Also, procedure difficulties resulted not only from the fact of removing the specific (HBP and LVP) lead but also from the presence of additional leads. And last but not least, this is presentation of a single very experienced centre. For this reason, the outcomes may not represent the overall safety and efficacy of TLE especially in patients with a long implant duration.

## Figures and Tables

**Table 1:** Characteristics of the study groups and main indications for lead extraction.

Patient-Related Risk Factors and Coexisting Indications for TLE (in 3897 Patients)	“Young” (≤40 mths) His Lead Extraction	“Old” (>40 mths) His Lead Extraction	“Young” (≤44 mths) LV Lead Extraction	“Old” (>44 mths) LV Lead Extraction	All TLEs with LV Lead Extraction	All TLEs with His Lead Extraction	TLE of Other than His and LV Leads
Group number/number of patients	Group 1 *n* = 13 mean ± SD *n* (%)	Group 2 *n* = 14 mean ± SD *n* (%) P: 1 vs. 2	Group 3 *n* = 127 mean ± SD *n* (%)	Group 4 *n* = 126 mean ± SD *n* (%) P: 3 vs. 4	Group 5 *n* = 253 mean ± SD *n* (%)	Group 6 *n* = 27 mean ± SD *n* (%) P: 5 vs. 6	Group 7 *n* = 3617 mean ± SD *n* (%) 5 vs. 7 6 vs. 7
Patient age during TLE [years]	68.38 ± 9.24	72.50 ± 7.43 *p* = 0.131	68.43 ± 10.58	69.30 ± 9.34 *p* = 0.560	68.87 ± 9.97	70.52 ± 8.45 *p* = 0.603	65.84 ± 16.05 *p* = 0.370 *p* = 0.167
Patient age at first system implantation [years]	64.92 ± 9.72	63.64 ± 8.03 *p* = 0.961	64.57 ± 11.49	61.60 ± 9.58 *p* = 0.012	63.09 ± 10.66	64.26 ± 8.73 *p* = 0.971	57.13 ± 17.51 *p* = 0.092 *p* = 0.001
Female	3 (23.08)	5 (35.71) *p* = 0.767	27 (21.26)	26 (20.63) *p* = 0.974	53 (20.95)	8 (29.63) *p* = 0.428	1424 (39.37) *p* = 0.001 *p* = 0.404
Coronary heart disease	10 (76.93)	6 (42.86) *p* = 0.159	63 (49.67)	55 (57.47) *p* = 0.410	118 (46.64)	15 (55.56) *p* = 0.498	2041 (56.43) *p* = 0.003 *p* = 0.917
NYHA FC III or IV	4 (30.77)	8 (57.14) *p* = 0.505	63 (49.61)	49 (38.89) *p* = 0.112	112 (44.27)	12 (44.44) *p* = 1.000	491 (13.58) *p* = 0.001 *p* = 0.001
LVEF [%]	43.31 ± 15.90	44.14 ± 11.03 *p* = 0.699	33.80 ± 13.95	33.05 ± 12.55 *p* = 0.929	33.43 ± 13.25	43.74 ± 13.33 *p* = 0.001	50.65 ± 14.82 *p* = 0.009 *p* = 0.001
Charlson co-morbidity index [points]	5.77 ± 3.42	6.57 ± 3.99 *p* = 0.980	6.54 ± 3.91	5.99 ± 3.53 *p* = 0.403	6.26 ± 3.73	6.19 ± 3.68 *p* = 0.972	4.81 ± 3.68 *p* = 0.030 *p* = 0.001
PADIT score [points]	4.69 ± 3.38	3.07 ± 2.84 *p* = 0.230	5.52 ± 2.81	5.98 ± 3.04 *p* = 0.154	5.75 ± 2.93	3.85 ± 3.16 *p* = 0.003	3.54 ± 2.82 *p* = 0.648 *p* = 0.001
Main indications for TLE—(primary/predominant)							
Infective endocarditis with or without pocket infection	4 (30.77)	5 (35.71) *p* = 0.892	60 (47.24)	63 (50.00) *p* = 0.755	123 (48.62)	9 (33.33) *p* = 0.190	722 (19.96) *p* = 0.001 *p* = 0.309
Local (isolated) pocket infection	2 (15.38)	0 (0.00) *p* = 0.430	20 (15.75)	14 (11.11) *p* = 0.370	34 (13.44)	2 (7.41) *p* = 0.130	330 (9.12) *p* = 0.001 *p* = 0.835
Mechanical lead damage (electrical failure)	2 (15.38)	2 (14.29) *p* = 0.644	9 (7.09)	21 (16.67) *p* = 0.031	30 (11.86)	4 (14.81) *p* = 0.891	1026 (28.37) *p* = 0.001 *p* = 0.179
Lead dysfunction (exit/ entry block, dislodgement, perforation, extracardiac pacing)	5 (38.46)	4 (28.57) *p* = 0.892	35 (27.56)	14 (11.11) *p* = 0.002	49 (19.37)	9 (33.33) *p* = 0.164	809 (22.37) *p* = 0.025 *p* = 0.516
Change of pacing mode/upgrading, downgrading and other non- infectious indications *	0 (0.00)	2 (14.29) *p* = 0.496	3 (2.36)	14 (11.11) *p* = 0.012	17 (6.72)	3 (11.11) *p* = 0.636	730 (20.18) *p* = 0.001 *p* = 0.201

TLE—transvenous lead extraction, LV (pacing) lead—a lead designed for pacing of the left ventricle, mths—months, *n*—number of patients/procedures, SD—standard deviation, NYHA FC—New York Heart Association (functional class), LVEF—left ventricular ejection fraction, * abandoned lead/prevention of abandonment (atrial fibrillation, redundant leads), threatening/potentially threatening lead (loops, free ends, left heart, lead-derived tricuspid valve defect), other (magnetic resonance imaging, cancer, painful pocket, loss of indications for pacing/implantable cardioverter defibrillator), and re-established venous access (symptomatic occlusion, superior vena cava syndrome, lead replacement/upgrading).

**Table 2:** System-related, history of pacing-related, and procedure-related risk factors for major complications and procedure complexity.

System-Related Risk Factors for TLE Complexity and Major Complications of TLE	“Young” (≤40 mths) His Lead Extraction	“Old” (>40 mths) His Lead Extraction	“Young” (≤44 mths) LV Lead Extraction	“Old” (>44 mths) LV Lead Extraction	All TLEs with LV Lead Extraction	All TLEs with His Lead Extraction	TLE of Other than His and LV Leads
Number of patients/group number	Group 1 *n* = 13 mean ± SD *n* (%)	Group 2 *n* = 14 mean ± SD *n* (%) P: 1 vs. 2	Group 3 *n* = 127 mean ± SD *n* (%)	Group 4 *n* = 126 mean ± SD *n* (%) P: 3 vs. 4	Group 5 *n* = 253 mean ± SD *n* (%)	Group 6 *n* = 27 mean ± SD *n* (%) P: 5 vs. 6	Group 7 *n* = 3617 mean ± SD *n* (%) 5 vs. 7 6 vs. 7
System and history of pacing							
Type of CIED							
Pacemaker	7 (53.85)	11 (78.57) *p* = 0.341	35 (27.56)	38 (30.16) *p* = 0.571	73 (28.85)	18 (66.67) *p* < 0.001	2678 (74.04) *p* = 0.001 *p* = 0.516
Implantable cardioverter defibrillator	0 (0.00)	0 (0.00) NA	0 (0.00)	3 (2.38) *p* = 0.243	3 (1.19)	0 (0.00) *p* = 0.679	845 (23.36) *p* = 0.001 *p* = 0.008
Cardiac resynchronisation cardioverter defibrillator	6 (46.15)	3 (21.43) *p* = 0.341	92 (72.44)	85 (67.46) *p* = 0.467	177 (69.96)	9 (33.33) *p* = 0.001	94 (2.60) *p* < 0.001 *p* < 0.001
Dwell time of oldest extracted lead [months]	41.31 ± 35.00	105.93 ± 42.40 <0.001	47.87 ± 45.20	93.24 ± 44.32 *p* < 0.001	70.46 ± 50.13	74.81 ± 50.47 *p* = 0.576	105.19 ± 77.55 *p* = 0.059 *p* = 0.001
Global lead dwell time before TLE [years]	6.24 ± 5.33	22.01 ± 9.33 <0.001	8.79 ± 7.78	21.68 ± 9.90 *p* < 0.001	15.21 ± 10.98	14.41 ± 11.00 *p* = 0.680	15.51 ± 13.15 *p* = 0.833 *p* = 0.369
HBP and LVP lead implant duration [months]	13.15 ± 8.38	92.93 ± 38.22 <0.001	21.94 ± 16.09	78.45 ± 26.98 *p* < 0.001	50.20 ± 35.96	54.52 ± 49.12 *p* = 0.568	NA NA NA
Abandoned lead before TLE	1 (7.69)	1 (7.14) *p* = 0.496	14 (11.02)	20 (15.87) *p* = 0.344	34 (13.44)	2 (7.41) *p* = 0.557	382 (10.56) *p* = 0.118 *p* = 0.828
Number of leads in the heart before TLE before TLE	2.38 ± 0.87	2.64 ± 0.50 *p* = 0.590	2.97 ± 0.62	3.10 ± 0.58 *p* = 0.175	3.04 ± 0.60	2.52 ± 0.70 *p* = 0.003	1.87 ± 0.68 *p* = 0.001 *p* = 0.001
Number of procedures before lead extraction	1.77 ± 0.60	2.08 ± 0.76 *p* = 0.294	1.91 ± 1.33	2.27 ± 0.98 *p* < 0.001	2.09 ± 1.18	1.92 ± 0.69 *p* = 0.830	1.84 ± 1.07 *p* = 0.146 *p* = 0.001
Scores for evaluation of risk factors for major complications or procedure complexity							
Safety score estimated risk of MC (risk of MC) [%]	0.60 ± 0.50	1.94 ± 1.96 *p* = 0.019	0.98 ± 2.72	2.08 ± 2.22 *p* < 0.001	1.54 ± 2.53	1.33 ± 1.61 *p* = 0.837	1.73 ± 2.98 *p* = 0.682 *p* = 0.697
2 and 3 EROS score [risk of MC]	4 (36.36)	4 (50.00) *p* = 0.767	65 (51.18)	68 (53.97) *p* = 0.853	133 (52.57)	16 (59.26) *p* = 0.646	1399 (38.70) *p* = 0.001 *p* = 0.047
MB score (need for advanced tools) [points]	1.85 ± 1.46	3.21 ± 0.58 *p* = 0.024	2.16 ± 1.39	3.45 ± 0.93 *p* < 0.001	2.80 ± 1.35	2.56 ± 1.28 *p* = 0.260	2.58 ± 1.24 *p* = 0.782 *p* = 0.005
LED index (predicted fluoroscopy time) [points]	5.54 ± 3.10	10.79 ± 3.36 *p* = 0.001	6.28 ± 4.16	10.32 ± 3.68 *p* < 0.001	8.29 ± 4.42	8.26 ± 4.15 *p* = 0.961	10.23 ± 6.53 *p* = 0.193 *p* = 0.001
Advanced LE (ALET—need for advanced TLE techniques) scale (3 and 4 points)	6 (46.15)	6 (42.86) *p* = 0.830	68 (53.64)	96 (76.19) *p* < 0.001	164 (64.82)	12 (44.44) *p* = 0.061	1217 (33.65) *p* = 0.001 *p* = 0.328
LECOM score (expected procedure complexity) [%]	16.19 ± 10.30	22.39 ± 15.70 *p* = 0.843	23.33 ± 18.49	30.88 ± 19.11 *p* < 0.001	27.09 ± 19.14	19.41 ± 13.50 *p* = 0.026	19.81 ± 18.29 *p* = 0.391 *p* = 0.001
TLE-related potential risk factors for major complications and procedure complexity							
Number of extracted leads per patient	2.23 ± 0.83	2.14 ± 0.86 *p* = 0.393	2.46 ± 1.01	2.75 ± 0.88 *p* = 0.044	2.60 ± 0.96	2.19 ± 0.83 *p* = 0.012	1.59 ± 0.66 *p* = 0.004 *p* = 0.001
Extraction of abandoned lead(s) (any)	1 (7.69)	1 (7.14) *p* = 0.496	13 (10.24)	18 (14.29) *p* = 0.453	31 (12.25)	2 (7.41) *p* = 0.668	351 (9.70) *p* = 0.228 *p* = 0.940
Extraction of passive- fixation lead (excluding LV lead)	7 (53.85)	10 (71.43) *p* = 0.585	125 (98.43)	124 (98.41) *p* = 0.620	249 (98.42)	17 (62.96) *p* < 0.001	1985 (54.88) *p* = 0.001 *p* = 0.518
Age of extracted HBP/LVP leads [months]	13.15 ± 8.38	92.93 ± 38.22 *p* = 0.000	21.94 ± 16.09	78.45 ± 26.98 *p* < 0.001	50.20 ± 35.96	54.52 ± 49.12 *p* = 0.568	NA NA NA
Oldest extracted lead per patient [months]	41.31 ± 35.00	105.93 ± 42.40 <0.001	45.68 ± 44.85	90.74 ± 41.08 *p* < 0.001	68.12 ± 48.50	74.81 ± 50.47 *p* = 0.458	103.49 ± 76.54 *p* = 0.076 *p* = 0.001

TLE—transvenous lead extraction, LV (pacing) lead—a lead designed for pacing of the left ventricle, mths—months, *n*—number of patients/procedures, SD—standard deviation HBP –His Bundle Pacing (lead), LVP—Left Ventricular Pacing (lead), CIED—cardiac implantable electronic devices, MC—major (TLE) complications, EROS—ELECTRa Registry Outcome Score, MB (score)—Mazzone-Bontempi (score), LED (index)—Lead Extraction Difficulty (index), ALET (score)—Advanced Lead Extraction Techniques (score), LECOM score—Lead Extraction COMplexity scoring system, NA—non-applicable.

**Table 3:** Extraction procedure complexity, major complications, and long-term outcomes.

Patient-Related Risk Factors and Coexisting Indications for TLE (in 3897 Patients)	“Young” (≤40 mths) His Lead Extraction	“Old” (>40 mths) His Lead Extraction	“Young” (≤44 mths) LV Lead Extraction	“Old” (>44 mths) LV Lead Extraction	All TLEs with LV Lead Extraction	All TLEs with His Lead Extraction	TLE of Other than His and LV Leads
Group number/number of patients	Group 1 *n* = 13 mean ± SD *n* (%)	Group 2 *n* = 14 mean ± SD *n* (%) P: 1 vs. 2	Group 3 *n* = 127 mean ± SD *n* (%)	Group 4 *n* = 126 mean ± SD *n* (%) P: 3 vs. 4	Group 5 *n* = 253 mean ± SD *n* (%)	Group 6 *n* = 27 mean ± SD *n* (%) P: 5 vs. 6	Group 7 *n* = 3617 mean ± SD *n* (%) 5 vs. 7 6 vs. 7
Procedure duration (sheath-to-sheath) [minutes]	7.85 ± 3.16	19.43 ± 17.82 *p* = 0.057	14.35 ± 17.97	25.38 ± 27.56 *p* < 0.001	19.85 ± 23.85	13.85 ± 14.08 *p* = 0.052	14.71 ± 23.25 *p* = 0.354 *p* = 0.001
Mean time of single lead extraction (sheath-to-sheath/number of extracted leads) [minutes]	3.87 ± 1.90	8.57 ± 6.97 *p* = 0.015	5.85 ± 5.94	9.31 ± 9.58 *p* < 0.001	7.57 ± 8.14	6.31 ± 5.63 *p* = 0.452	9.02 ± 13.00 *p* = 0.161 *p* = 0.043
Time of single HBP/LVP lead extraction [minutes]	2.15 ± 2.23	4.14 ± 1.96 *p* = 0.021	2.07 ± 2.24	4.05 ± 5.19 *p* < 0.001	3.05 ± 4.10	3.19 ± 2.29 *p* = 0.868	NA NA NA
Removal of HBP/LVP lead with simple traction	4 (30.77)	0 (0.00) *p* = 0.025	63 (49.61)	10 (7.94) *p* < 0.001	73 (28.97)	4 (14.81) *p* = 0.185	NA NA NA
Extraction of HBP/LVP lead using mechanical dilation	9 (69.23)	14 (100.0) *p* = 0.088	64 (50.39)	116 (91.27) *p* < 0.001	179 (70.75)	23 (85.19) *p* = 0.127	NA NA NA
Number of unexpected procedure difficulties (“technical problems”) per patient	0.00 ± 0.00	1.75 ± 0.96 *p* = 0.045	1.57 ± 0.85	1.50 ± 0.67 *p* = 0.049	1.52 ± 0.72	1.75 ± 0.96 *p* = 0.800	1.45 0.77 *p* = 0.753 *p* = 0.908
CID-TLE score [points]	0.00	0.93 ± 1.33 *p* = 0.004	0.43 ± 0.94	0.98 ± 1.23 *p* < 0.001	0.70 ± 1.12	0.48 ± 1.05 *p* = 0.620	0.54 ± 1.12 *p* = 0.773 *p* = 0.012
CID-TLE score ≥ 2 points	0 (0.00)	5 (35.71) *p* = 0.059	22 (17.32)	49 (38.89) *p* < 0.001	71 (28.06)	5 (18.52) *p* = 0.405	662 (18.30) *p* = 0.001 *p* = 0.825
Use of additional tools							
Evolution (old and new) or TightRail	0 (0.00)	1 (7.14) *p* = 0.970	1 (0.79)	2 (1.59) *p* = 1.000	3 (1.19)	1 (3.70) *p* = 0.845	61 (1.69) *p* = 0.001 *p* = 0.952
Metal sheath, lasso-basket catheter	0 (0.00)	3 (21.43) *p* = 0.247	8 (6.30)	22 (17.46) *p* = 0.011	30 (11.86)	3 (11.11) *p* = 0.842	499 (13.80) *p* = 0.440 *p* = 0.902
TLE efficacy and complications							
Major complications (any)	0 (0.00)	0 (0.00) NA	1 (0.79)	1 (0.79) *p* = 0.481	2 (0.79)	0 (0.00) *p* = 0.460	78 (2.16) *p* = 0.212 *p* = 0.917
Haemopericardium	0 (0.00)	0 (0.00) NA	1 (0.79)	0 (0.00) *p* = 0.997	1 (0.40)	0 (0.00) *p* = 0.171	45 (1.24) *p* = 0.366 *p* = 0.771
Rescue cardiac surgery	0 (0.00)	0 (0.00) NA	1 (0.79)	1 (0.79) *p* = 1.000	2 (0.79)	0 (0.00) *p* = 0.460	38 (1.05) *p* = 0.941 *p* = 0.678
Tricuspid valve damage during TLE (severe)	0 (0.00)	0 (0.00) NA	0 (0.00)	0 (0.00) NA	0 (0.00)	0 (0.00) NA	25 (0.69) *p* = 0.357 *p* = 0.461
Death, procedure-related (intra-, post-procedural)	0 (0.00)	0 (0.00) NA	1 (0.79)	0 (0.00) *p* = 0.997	1 (0.40)	0 (0.00) *p* = 0.171	5 (0.14) *p* = 0.859 *p* = 0.847
Partial radiographic success (retained tip or <4 cm lead fragment)	0 (0.00)	0 (0.00) NA	0 (0.00)	8 (6.35) *p* = 0.012	8 (3.16)	0 (0.00) *p* = 0.742	145 (4.01) *p* = 0.616 *p* = 0.573
Procedural success	13 (100.0)	14 (100.0) NA	125 (98.43)	118 (93.65) *p* = 0.104	243 (96.05)	27 (100.0) *p* = 0.613	3444 (95.22) *p* = 0.654 *p* = 0.978
Mortality after TLE (death for each time interval)							
All model log rank *p* < 0.001							
Survivors during follow-up	7 (53.85)	8 (57.14) *p* = 0.830	47 (37.01)	59 (46.83) *p* = 0.146	106 (41.90)	15 (55.56) *p* = 0.247	2264 (62.59) *p* = 0.001 *p* = 0.580
Deaths during follow-up	6 (45.15)	6 (42.86) *p* = 0.830	80 (62.99)	67 (53.18) *p* = 0.146	147 (58.10)	12 (44.44) *p* = 0.247	1353 (37.41) *p* = 0.001 *p* = 0.580
Perioperative death (first two days)	0 (0.00)	0 (0.00) NA	1 (0.79)	0 (0.00) *p* = 0.997	1 (0.40)	0 (0.00) *p* = 0.171	13 (0.36) *p* = 0.653 *p* = 0.191
Intra-hospital death (3rd–30th day)	2 (15.38)	0 (0.00) *p* = 0.430	5 (3.94)	4 (3.17) *p* = 0.990	9 (3.56)	2 (7.41) *p* = 0.647	45 (1.24) *p* = 0.006 *p* = 0.049
		Log rank: *p* = 0.132		Log rank: *p* = 0.522		Log rank: *p* = 0.325	Log rank: *p* = 0.005 *p* = 0.010
Death within the first year of follow-up (31–365 days)	3 (23.08)	1 (7.14) *p* = 0.534	23 (18.11)	19 (15.08) *p* = 0.632	42 (16.60)	4 (14.81) *p* = 0.972	217 (6.00) *p* = 0.001 *p* = 0.132
One-year follow-up		Log rank: *p* = 0.022		Log rank: *p* = 0.349		Log rank: *p* = 0.695	Log rank: *p* < 0.001 *p* = 0.002
Death at 1 to 3 years of follow-up (366–1095 days)	1 (7.69)	3 (21.43) *p* = 0.644	22 (17.32)	21 (16.67) *p* = 0.977	43 (17.00)	4 (14.81) *p* = 0.986	306 (8.46) *p* = 0.000 *p* = 0.405
Three-year follow-up		Log rank: *p* = 0.062		Log rank: *p* = 0.374		Log rank: *p* = 0.654	Log rank: *p* < 0.001 *p* < 0.001
Late death, at >3 years (>1095 days) of follow-up	0 (0.00)	2 (14.29) *p* = 0.496	29 (22.83)	23 (18.25) *p* = 0.456	52 (20.55)	2 (7.41) *p* = 0.165	772 (21.34) *p* = 0.828 *p* = 0.135
Death during follow-up		Log rank: *p* = 0.099		Log rank: *p* = 0.354		Log rank: *p* = 0.780	Log rank: *p* < 0.001 *p* < 0.001
Follow-up [days]	940.8 ± 1204	1334 ± 1088 *p* = 0.148	1458 ± 1376	1407 ± 1239 *p* = 0.882	1153 ± 1135	1432 ± 1307 *p* = 0.337	2122 ± 1469 *p* < 0.001 *p* < 0.001
The length of the FU period for survivors [days]	1303.86 ± 1451.79	1559 ± 1346 *p* = 1.000	2095.11 ± 1548.34	1973 ± 1361 *p* = 0.629	2027 ± 1441	1440 ± 1351 *p* = 0.462	2481 ± 1516.5 *p* < 0.001 *p* < 0.001
The length of the FU period for non-survivors [days]	284.3 ± 314.7	906.0 ± 646.0 *p* = 0.020	1072 ± 1137	929.0 ± 919.8 *p* = 0.832	1007 ± 1043	595.2 ± 583.2 *p* = 0.243	1578 ± 1251.8 *p* < 0.001 *p* = 0.003

TLE—transvenous lead extraction, LV (pacing) lead—a lead designed for pacing of the left ventricle, mths—months, *n*—number of patients/procedures, SD—standard deviation, HBP—His Bundle Pacing (lead), LVP—Left Ventricular Pacing (lead), CID-TLE—Complex Indicator of the Difficulty of the TLE, NA—non-applicable.

**Table 4:** Differences between survivors and non-survivors of His lead extraction.

His Pacing Lead Extraction Group Potential Risk Factors for Shorter Survival	Survivors *n* = 15	Non- Survivors *n* = 12	*p* Mann–Whitney U Test Chi2
Female	7 (46.67)	2 (16.67)	0.218
Patient age [years]	71.25 ± 7.82	69.83 ± 10.14	0.707
Left ventricular ejection fraction [%]	50.18 ± 12.55	40.33 ± 13.71	0.117
Congestive heart failure	5 (33.33)	7 (58.33)	0.553
CHA_2_DS_2_–VASc score [points]	3.50 ± 1.51	3.25 ± 1.29	0.729
Charlson co-morbidity index [points]	6.33 ± 4.03	5.25 ± 3.31	0.817
PADIT [points]	3.33 ± 3.03	4.33 ± 3.55	0.525
Oldest extracted lead per patient [years]	7.65 ± 4.26	4.40 ± 3.18	0.057
Infectious indications for TLE	5 (3.33)	6 (50.00)	0.630
ICD lead presence before TLE	1 (6.67)	6 (60.00)	0.035

CHA_2_DS_2_–VASc—(Congestive heart failure, Hypertension, Age 75 years and older, Diabetes, Stroke, Vascular disease, Age 65 to 74 years, Sex Category (female))—ischemic risk stroke score in patients with atrial fibrillation, PADIT—(Prevention of Arrhythmia Device Infection Trial) risk score of device infection, TLE—transvenous lead extraction, ICD—implantable cardioverter defibrillator.

## Data Availability

Readers can access the data supporting the conclusions of the study at www.usuwanieelektrod.pl.
